# Association Between Current of Injury Magnitude and Intraoperative R-Wave Amplitude Fluctuation After Pacing Lead Implantation

**DOI:** 10.1016/j.jacasi.2026.01.030

**Published:** 2026-04-11

**Authors:** Yoshitake Oshima, Satoshi Oka, Kohei Ishibashi, Toshihiro Nakamura, Yuichiro Miyazaki, Akinori Wakamiya, Nobuhiko Ueda, Kenzaburo Nakajima, Tsukasa Kamakura, Mitsuru Wada, Yuko Inoue, Koji Miyamoto, Satoshi Nagase, Takeshi Aiba, Kengo Kusano

**Affiliations:** a Department of Cardiovascular Medicine, National Cerebral and Cardiovascular Center, Suita, Osaka, Japan; b Department of Cardiology, Gifu Prefectural General Medical Center, Gifu, Gifu, Japan; c Department of General Internal Medicine 3, Kawasaki Medical School General Medical Center, Okayama, Japan

**Keywords:** active fixation lead, current of injury, pacemaker, R-wave amplitude, electrogram

## Abstract

**Background:**

Pacing threshold adequacy and lead stability can be predicted from the magnitude of the current of injury (COI) immediately after active fixation. However, intraoperative R-wave amplitude often fluctuates despite sufficient COI.

**Objectives:**

The authors aimed to assess whether R-wave amplitude fluctuations are associated with the COI magnitude and can be predicted from early electrogram (EGM) patterns.

**Methods:**

EGMs of 109 cases of right ventricular pacing on the septum were continuously recorded from implantation to generator connection. R-wave amplitude fluctuation patterns were categorized as Dipper type (transient decrease followed by an increase), Riser type (continuous increase), and Other type. The primary outcome was a favorable R-wave amplitude increase, defined as the final value exceeding the initial value.

**Results:**

Among the 109 cases, 65 (60%) exhibited Dipper-type and 41 (38%) showed Riser-type R-wave fluctuation patterns. In the Dipper-type patterns, initial EGMs showed R-wave amplitude < COI, which subsequently shifted to R-wave amplitude > COI patterns. Favorable amplitude increases occurred in 34 (52%) of the Dipper-type cases and were associated with shorter times to the lowest R-wave amplitude, with 6 minutes being identified as optimal for prediction (sensitivity: 0.82, specificity: 0.61). All the Riser-type patterns exhibited favorable amplitude increases, with consistent R-wave amplitude > COI patterns.

**Conclusions:**

Most intraoperative R-wave amplitude fluctuations in successful lead implantations follow Dipper- or Riser-type patterns. Favorable R-wave amplitude increases can be predicted within 6 minutes post fixation using early EGM patterns.

Paced QRS duration is associated with functional and clinical prognosis in pacemaker recipients.^1^^,^^2^ Recently, conduction-system pacing has gained attention.^3^^,^^4^ Delivery catheter and stylet lumen-less active fixation lead (C315 guiding sheath and model 3830 lead; Medtronic Inc) are useful for attaining accurate right ventricular (RV) septal pacing and shorter QRS duration.^5^^,^^6^ As this lead has a fixed helix, its implantation into the myocardium causes tissue injury, leading to a phenomenon known as the current of injury (COI); therefore, adequate pacing thresholds and local myocardial potential amplitudes may not be obtained immediately. The COI is recognized on the implanted lead electrogram (EGM) as an ST-segment elevation. The stability of the lead and the pacing threshold adequacy can be predicted from the magnitude of the COI.^7^^,^^8^ Contrastingly, we have often observed a decrease in the intraoperative R-wave amplitude automatically measured by the device programmer from the value immediately after the implantation and have considered whether lead repositioning should be performed while a favorable COI prevalence is obtained. This phenomenon could be hypothesized as follows: 1) the device programmer initially misidentifies the very high COI magnitude as “R-wave amplitude;” and 2) it takes time for COI magnitude withdrawal and to detect the true local myocardial potential. However, little is known because Medtronic Inc is unwilling to disclose the pacing system analyzer filter settings.^9^

In this study, we aimed to clarify whether intraoperative R-wave amplitude fluctuation is associated with the magnitude of COI and whether it is possible to predict its adequacy from the EGM immediately after the active lead fixation.

## Methods

### Study population

We retrospectively studied consecutive cases of model 3830 active fixation lead implantation to the RV septum using a C315 guiding catheter performed at the National Cerebral and Cardiovascular Center, Suita, Japan, from April 2023 to May 2024. The indications for pacemaker implantation were based on Japanese guidelines.^10^ Conduction-system pacing attempted implantations were excluded to minimize the bias due to the differences in lead depth, COI magnitude,^11^ and time from screw-in to start of bipolar R-wave amplitude recording compared with RV pacing on the septum.

This study was approved by the Institutional Research Board of the National Cerebral and Cardiovascular Centre, Suita, Japan (M26-150-19) and was conducted in accordance with the 1975 Declaration of Helsinki.

### Consent

All patients provided written informed consent before device implantation.

### Implantation procedure and data collection

Intracardiac devices were implanted in a standard manner. The RV lead was positioned on the RV septum using a guiding catheter. The bipolar RV lead EGM was continuously recorded (25 mm/s, 1.0 mV/mm) immediately after active fixation until the connection of the generator. The R-wave amplitude values were automatically measured using a programmer (Care Link Smart Sync; Medtronic Inc). The pacing threshold, lead impedance, and COI magnitude were measured manually, at least immediately before and after lead fixation and at the end of the procedure, with additional measurements as needed. Lead implantation success criteria were as follows: 1) final R-wave amplitude value >4 mV; 2) pacing capture threshold <1.5 V @ 0.4 ms; 3) lead impedance within 400 to 1,200 Ohms;^12^ and 4) the presence of an adequate COI, that is, a maximum amplitude of the elevated ST-segment of the EGM >2 mV.^13^ Lead implantation was considered unsuccessful if: 1) any one of the preceding success criteria was not met; 2) the operator judged that lead replacement was necessary; and 3) intraoperative lead dislodgement was observed.

### Classification of R-wave amplitude fluctuation

**Figure 1:**
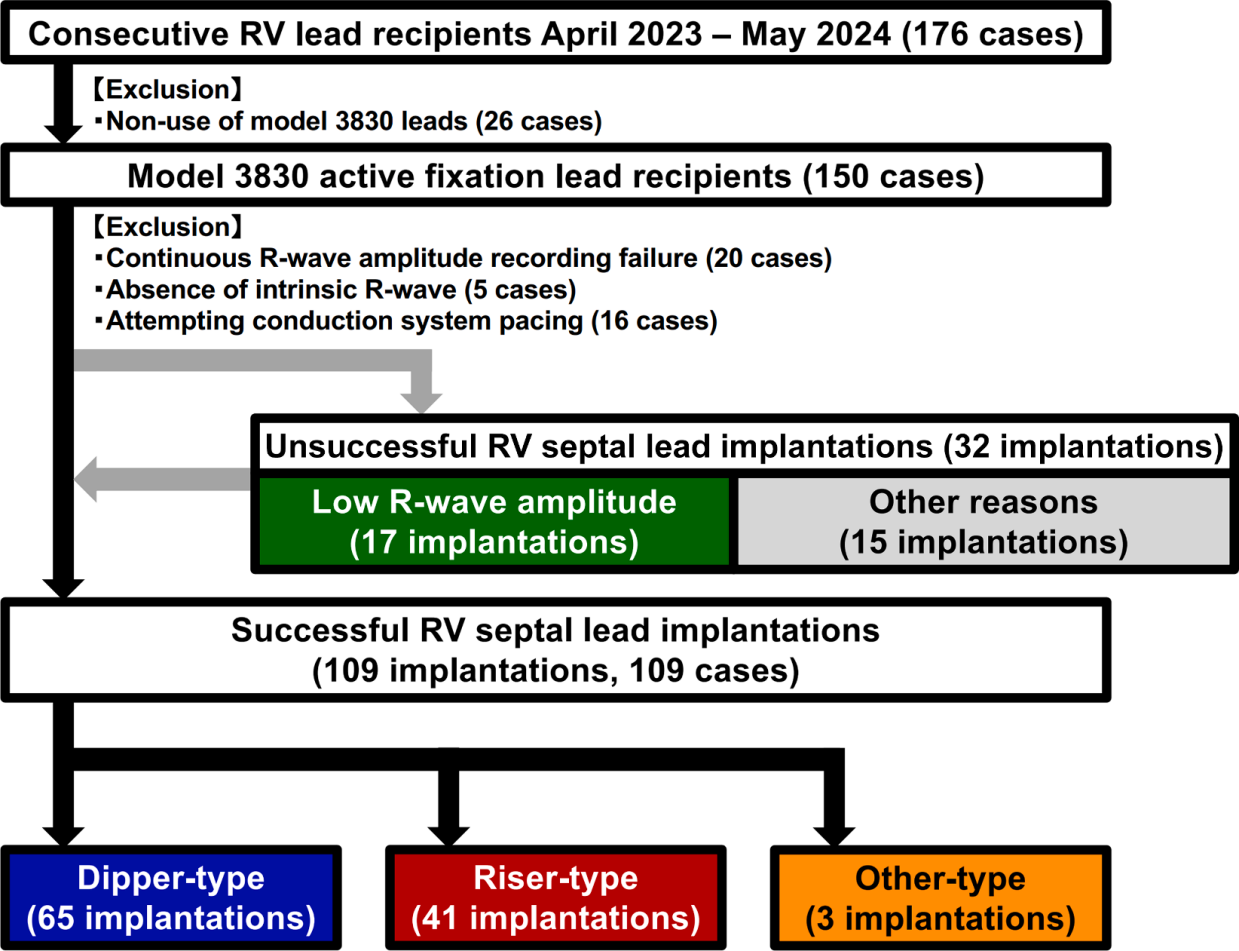
Flow Chart of the Patients Included in the Analyses RV = right ventricular.

Patterns of R-wave amplitude fluctuation were classified into 3 types: 1) the “Dipper type,” in which R-wave amplitude initially decreased transiently to the minimum value and then increased thereafter; 2) the “Riser type,” in which R-wave amplitude initially showed an upward trend (no decline) over the first 5 minutes after active fixation, with continued increase thereafter; and 3) “Other type” (Figure 1). In the Dipper type, screw-in-to-bottom time (SBT), defined as the time taken from the active lead fixation to R-wave amplitude reaching the minimum value, was measured and evaluated.

### Clinical outcome

The primary outcome was a favorable R-wave amplitude increase, defined as successful lead implantation, with the final recorded R-wave amplitude exceeding that measured immediately after lead fixation.

### Statistical analysis

A complete case analysis was performed, and no imputation was applied for missing data. Normally distributed continuous variables are expressed as means and standard deviations. The Kolmogorov-Smirnov test was used to detect variables that were not normally distributed, which are expressed as medians and IQRs. Student’s *t*-test or the Mann-Whitney *U* test was used to compare the differences between the 2 distribution groups, as appropriate. Categorical variables were compared using Fisher’s exact test. The optimal cutoff value of SBT for predicting the primary outcome was determined using a receiver operating characteristic (ROC) curve analysis. A logistic regression model was used to identify predictors for the primary outcome. Variables considered clinically relevant to intraoperative R-wave amplitude fluctuation were first examined using univariate logistic regression. Among these, only those that were statistically significant in the univariate analysis were subsequently entered as covariates into the multivariate model. All statistical tests were 2 tailed. *P* < 0.05 was considered significant. All statistical analyses were performed using EZR software (version 1.54, Saitama Medical Center, Jichi Medical University), a graphical user interface of R (The R Foundation for Statistical Computing).

## Results

### Successful RV lead implantation

Of the 176 consecutive patients who underwent RV lead implantation, 109 patients who underwent RV pacing on the septum (mean age 75 ± 13 years, 52 women, 17 patients with cardiomyopathies) were enrolled and evaluated. A total of 67 patients were excluded for reasons such as non-use of model 3830 lead (n = 26), continuous R-wave amplitude recording failure (n = 20), absence of intrinsic R-wave (n = 5), and attempting conduction-system pacing (n = 16). Although 32 implantations were initially unsuccessful, successful RV lead implantation was finally achieved in all 109 participants. Of the 32 unsuccessful lead implantations, 17 were considered failures because of inadequate R-wave amplitude (<4 mV) and 15 were attributed to other reasons, that is, inadequate lead pacing threshold or impedance (n = 11), inappropriate lead implantation site (n = 2), and intraoperative lead dislodgement (n = 2). The mean number of active fixations was 1.2 ± 0.6 per case. The success rate of the first active fixation was 85%.

### R-wave amplitude fluctuation patterns in successful lead implantation

Of the 109 successful lead implantations, 65 (60%) and 41 (38%) were classified as the Dipper- and Riser-type R-wave amplitude fluctuation patterns, respectively. The remaining 3 (3%) implantations were classified as the Other-type pattern.

### Dipper type of R-wave amplitude fluctuation

**Figure 2:**
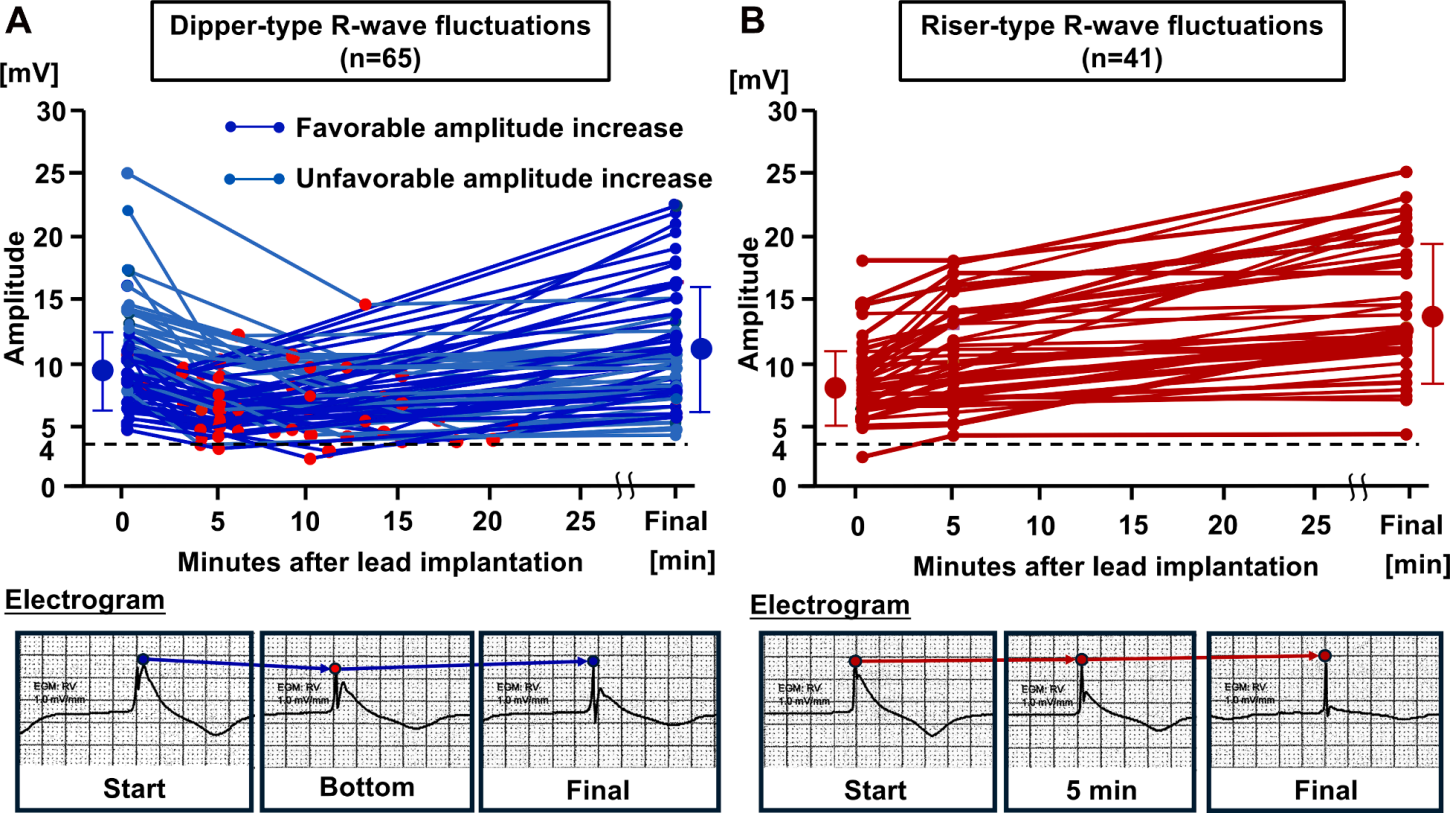
Intraoperative R-Wave Amplitude Fluctuation Patterns of Dipper and Riser Types (A) The Dipper-type R-wave amplitude transitions of the successful right ventricular pacing lead implantations on the septum. The R-wave amplitude initially decreased to the minimum value (red dot) and then increased. The cases with and without favorable amplitude increase are shown as blue and light blue lines, respectively. (B) The Riser-type R-wave amplitude transitions of the successful right ventricular pacing lead implantations on the septum. The bottom panels show the typical transitions of the bipolar electrograms recorded on the right ventricular leads in representative cases of each type. The blue and red dots indicate the highest amplitude points in each phase.

For the Dipper type, the minimum value was lower than that measured immediately after lead fixation; however, this value recovered over time and eventually reached an acceptable value (Figure 2A). The median SBT was 5 (IQR: 5-10) minutes, and then the R-wave amplitude improved to a mean of 1.7 ± 0.6 times higher than the minimum value. The EGM immediately after active fixation showed a pattern of initial R-wave amplitude < maximum COI magnitude, which gradually changed to R-wave amplitude > maximum COI magnitude by the withdrawal of the COI and an increase of the R-wave amplitude over time (Figure 2A). As an alternative EGM pattern, 6 cases (9%) showed a negative R-wave deflection (Supplemental Figure 1).

### Riser-type of R-wave fluctuation pattern after active lead fixation

For the Riser type, a pattern of an acceptable R-wave amplitude and R-wave amplitude > maximum COI magnitude pattern was consistently recorded on EGM for all implantations (Figure 2B). As an alternative EGM pattern, 4 cases (11%) showed a negative R-wave deflection (Supplemental Figure 1).

### Other type of R-wave fluctuation pattern after active lead fixation

Only 3 successful implantations could not be classified as Dipper or Riser types and were defined as the Other type. In this type, the R-wave amplitude value showed a slight decrease after lead fixation and reached a plateau while remaining within an acceptable range. The EGM morphology initially showed R-wave amplitude < maximum COI magnitude, which rapidly shifted to R-wave amplitude > maximum COI magnitude over time (Supplemental Figure 2A).

### Comparison of baseline characteristics and lead parameters between Dipper and Riser types

A comparison of the patient characteristics and lead parameters of Dipper, Riser, and Other types is presented in Table 1. No statistically significant differences in patient backgrounds were observed between the groups. In terms of lead parameters, although no statistically significant difference in lead impedance (786 ± 125 Ohms vs 756 ± 103 Ohms; *P* = 0.42) and pacing threshold (1.3 ± 0.7 V vs 1.3 ± 0.9 V; *P* = 0.83) were observed, the manually measured COI magnitude (13.9 ± 4.2 mV vs 9.5 ± 4.4 mV; *P* < 0.001) and R-wave amplitude (10.3 ± 3.8 mV vs 8.5 ± 3.1 mV; *P* = 0.02) were significantly higher in the Dipper-type fluctuation than in the Riser-type fluctuation immediately after the lead implantation. Furthermore, the final value of R-wave amplitude was significantly lower in the Dipper-type fluctuation than in the Riser-type fluctuation (10.7 ± 4.3 mV vs 14.6 ± 5.4 mV; *P* < 0.001).

**Table 1:** Patient Characteristics and Lead Parameters in Successful Right Ventricular Pacing Lead Implantations on the Septum

	All (N = 109)	Dipper Type (n = 65)	Riser Type (n = 41)	Other Type (n = 3)	*P* Value
Age, y	75 ± 13	75 ± 12	77 ± 15	65 ± 15	0.27
Female	52 (48)	27 (42)	24 (59)	1 (33)	0.21
Cardiomyopathy	17 (16)	8 (12)	8 (20)	1 (33)	0.42
Pacing indication					0.78
Sick sinus syndrome	34 (31)	23 (35)	10 (24)	1 (33)	
Atrioventricular block	65 (60)	36 (55)	27 (66)	2 (67)	
Others	10 (9)	6 (9)	4 (10)	0 (0)	
CIED types					0.15
Pacemaker	98 (90)	61 (94)	35 (85)	2 (67)	
CRT-P	11 (10)	4 (6)	6 (15)	1 (33)	
CIED chamber					0.25
Single chamber	8 (7)	7 (11)	1 (2)	0 (0)	
Dual chamber	101 (93)	58 (89)	40 (98)	3 (100)	
Lead parameters					
Immediately after implantation					
Lead impedance, ohms	774 ± 116	786 ± 125	756 ± 103	767 ± 44	0.42
Manually measured COI magnitude, mV^a^	12.2 ± 4.7	13.9 ± 4.2	9.5± 4.4	12.7 ± 4.2	<0.001
R-wave amplitude, mV^a^	9.7 ± 3.6	10.3 ± 3.8	8.5 ± 3.1	11.8 ± 1.2	0.02
Pacing threshold, V @ 0.4 ms	1.3 ± 0.8	1.3 ± 0.7	1.3 ± 0.9	1.1 ± 0.6	0.83
Final values					
Lead impedance, ohms	693 ± 100	707 ± 106	675 ± 89	646 ± 50	0.20
Manually measured COI magnitude, mV	4.1 ± 3.3	4.6 ± 3.3	3.2 ± 3.0	4.8 ± 2.8	0.08
R-wave amplitude, mV^a^	12.1 ± 5.1	10.7 ± 4.3	14.6 ± 5.4	8.8 ± 2.2	<0.001
Pacing threshold, V @ 0.4 ms	0.6 ± 0.2	0.6 ± 0.2	0.6 ± 0.3	0.5 ± 0.2	0.89
Favorable amplitude increase^a^	75 (69)	34 (52)	41 (100)	0 (0)	<0.001

Values are mean ± SD for normally distributed data and median (IQR) for non-normally distributed data, or n (%).

CIED = cardiovascular implantable electronic device; COI = current of injury; CRT-P = cardiac resynchronization therapy pacemaker.

a All statistical tests between the Dipper and Riser types were 2-tailed, and *P* < 0.05 was considered significant.

### Favorable R-wave amplitude increase

**Figure 3:**
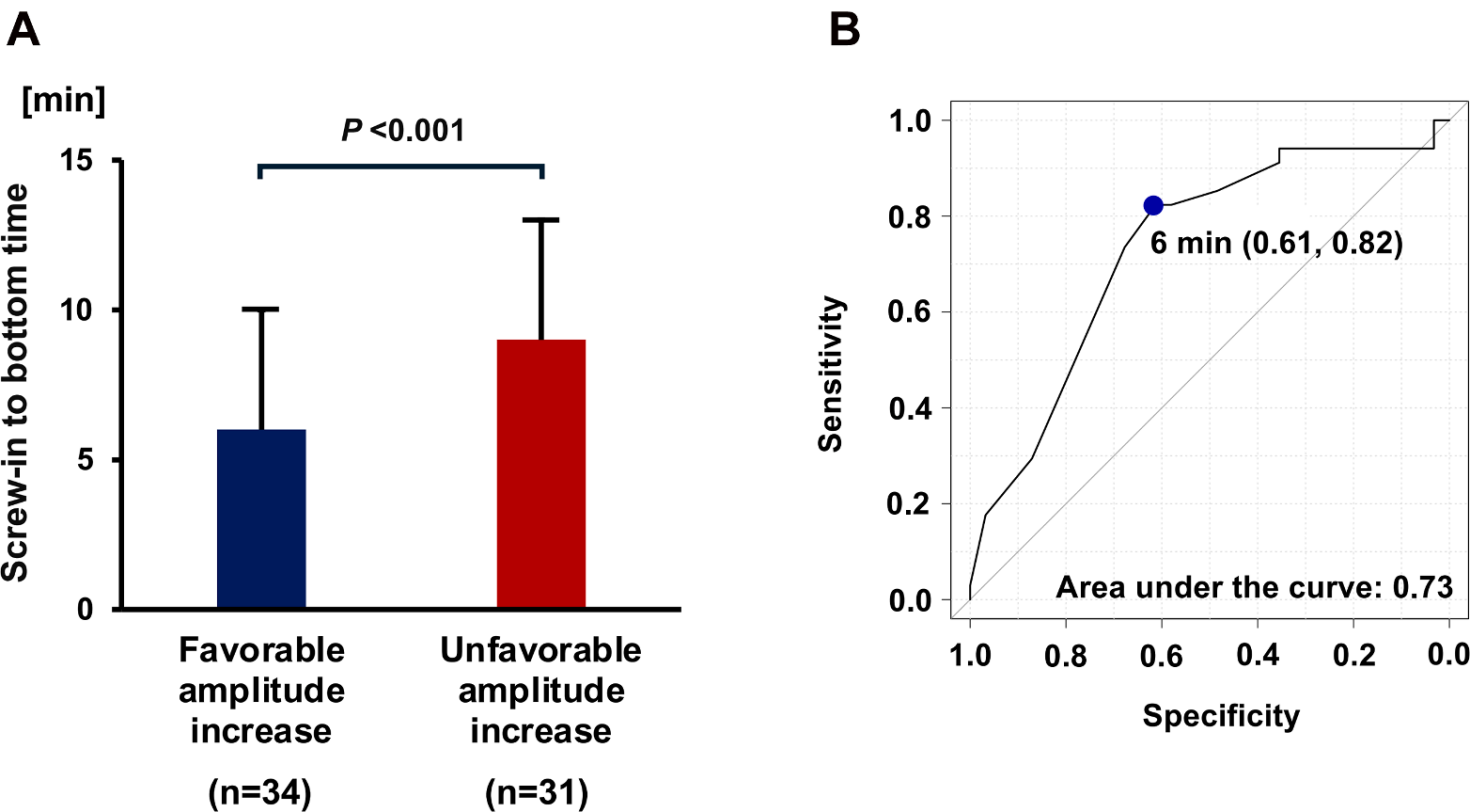
Relationship Between Favorable Amplitude Increase and Screw-In-To-Bottom Time in Dipper Type (A) Dipper-type fluctuations with a favorable amplitude increase show significantly shorter screw-in-to-bottom time. (B) The cutoff value of screw-in-to-bottom time for predicting a favorable amplitude increase is 6 minutes.

Among the Dipper-type implantations (n = 65), 34 implantations (52%) showed favorable R-wave amplitude increase and had significantly shorter SBT than the remaining implantations (6 ± 4 minutes vs 9 ± 4 minutes, *P* < 0.001) (Figure 3A). The ROC curve analysis revealed that the optimal cutoff value of SBT for predicting a favorable R-wave amplitude increase was 6 minutes (area under the curve: 0.73; 95% CI: 0.61-0.85; sensitivity: 0.82; 95% CI: 0.58-0.88; specificity: 0.61; 95% CI: 0.50-0.83) (Figure 3B). Contrastingly, a favorable amplitude increase was obtained in all the Riser-type implantations but not in all the Other-type implantations. Multivariate logistic regression analysis showed that SBT <6 minutes (OR: 15.3; 95% CI: 4.54-51.3; *P* < 0.001) and post-screw R-wave amplitude (OR: 0.24; 95% CI: 0.10-0.58; *P* = 0.001) were associated with a favorable R-wave amplitude increase (Table 2).

**Table 2:** Univariate and Multivariate Logistic Regression Analyses for Predicting Favorable R-Wave Amplitude Increases

	Univariate Analysis			Multivariate Analysis		
	OR	95% CI	*P* Value	OR	95% CI	*P* Value
Age, per 1 y	1.02	0.99–1.05	0.27		—	
Female	1.04	0.46–2.34	0.93		—	
Cardiomyopathy	1.1	0.36–3.43	0.86		—	
Post-screw R-wave amplitude, per 3.6 mV^a^	0.2	0.10–0.41	<0.001	0.24	0.10–0.58	0.001^a^
Post-screw COI magnitude, per 4.7 mV	0.44	0.28–0.70	<0.001	0.6	0.23–1.54	0.29
Post-screw lead impedance, per 116 ohms	0.49	0.27–0.90	0.02	0.57	0.29–1.12	0.10
Screw-in-to-bottom time <6 min^a^	17.6	6.38–48.5	<0.001^a^	15.3	4.54–51.3	<0.001^a^

The ORs and 95% CIs for continuous variables, excluding age, were estimated using a 1-SD increase.

Abbreviations as in Table 1.

a All statistical tests were 2-tailed, and *P* < 0.05 was considered significant.

### R-wave amplitude fluctuation pattern in unsuccessful implantation due to low amplitude

Of the 32 unsuccessful lead implantations, 17 were failures because of inadequate R-wave amplitude. Two types of cases were encountered. The first type of cases were those in which the R-wave amplitude immediately after implantation was below the success criterion (n = 6). In these cases in which immediate lead repositioning was considered, subsequent EGM changes could not be recorded. The second type of cases were those in which the R-wave amplitude was adequate immediately after implantation but decreased over time (n = 11). In these cases, EGM changes were observed for a period after the lead implantation. In contrast with successful cases, however, no increase in the R-wave amplitude was observed in the failed cases despite COI regression over time (Supplemental Figure 2B).

## Discussion

### Main findings

In this study, we continuously recorded and evaluated the intraoperative R-wave amplitude fluctuations after active fixation lead implantation. We found that more than 97% of the intraoperative R-wave amplitude fluctuations could be classified as Dipper or Riser types. The mechanism of the R-wave amplitude fluctuation may be related to the competition between the R-wave amplitude and the COI magnitude. EGM patterns immediately after RV lead implantation and subsequent R-wave amplitude trends for 5 to 6 minutes after implantation may predict a favorable R-wave amplitude increase (Central Illustration).

**Central Illustration:**
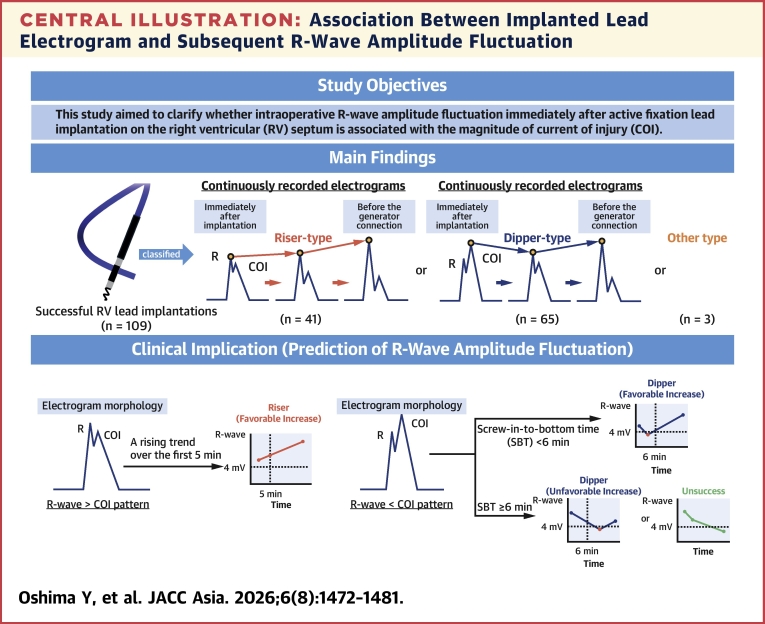
Association Between Implanted Lead Electrogram and Subsequent R-Wave Amplitude Fluctuation Most intraoperative R-wave amplitude fluctuation patterns immediately after successful lead implantation could be classified into Dipper or Riser types. Electrogram patterns immediately after right ventricular lead implantation and subsequent R-wave amplitude trends for 5 to 6 minutes after implantation may predict a favorable R-wave amplitude increase.

### Mechanism of R-wave amplitude fluctuation

To our knowledge, no study has investigated the temporal changes in R-wave amplitude following active lead fixation to the human heart through continuous monitoring. De Buitlier et al^14^ recorded R-wave amplitudes for 30 minutes after ventricular lead implantation; however, their recordings were intermittent and showed a consistent increase over time. Contrastingly, we used continuous EGM monitoring following active lead fixation and demonstrated that most successfully implanted cases exhibited either a Dipper-type fluctuation, characterized by a temporary R-wave amplitude decrease, or a Riser-type fluctuation, marked by a consistent R-wave amplitude increase.

We speculate that the potential mechanisms behind intraoperative R-wave fluctuations are as follows. 1) The active fixation leads initially cause trauma to the myocardial tissue.^7^ 2) Consequently, adequate local myocardial potential amplitude (ie, true R wave) cannot be obtained, and maximum COI magnitude is recorded immediately after the active fixation.^14^ 3) If a relatively high COI magnitude compared with the local myocardial amplitude is recorded, the device programmer can initially misidentify the COI as “R-wave” amplitude and then deem local myocardial potential amplitude to be an R-wave due to the decrease in COI magnitude^7^ over time (the Dipper type). Hence, there is a mismatch between the programmer-measured R-wave and the true R-wave initially, but these values become consistent as the COI magnitude diminishes. 4) If a relatively high local myocardial amplitude compared with the COI magnitude is recorded, the device programmer can consistently identify the true R wave and finally recognize the adequate value (the Riser type).

In the Dipper type, the EGM immediately after active fixation, showing an R-wave amplitude < COI magnitude pattern morphology supports the aforementioned hypothesis. Furthermore, a previous study that continuously monitored EGM after active fixation of fully rotated model 3830 pacing leads in rabbit hearts also demonstrated an alternation from R-wave amplitude < COI magnitude pattern to R-wave amplitude > COI magnitude pattern morphology.^15^ Because the withdrawal of COI magnitude was reported to be within 5 minutes,^8^ the SBT can be shorter than 5 to 6 minutes if the local myocardial potential amplitude is sufficient to obtain a favorable amplitude increase.

In the Riser type, the EGM immediately after active fixation displayed an R-wave amplitude > COI magnitude pattern, and local myocardial amplitude detection consistently captured the true R-wave peak. Thus, a favorable R-wave amplitude increase with 0 minutes of SBT was obtained in all the Riser-type pattern patients. In patients with an alternative EGM pattern with negative R-wave deflection, the same mechanisms could be considered (Supplemental Figure 1).

### Prediction of favorable R-wave amplitude increase and clinical implications

The findings of this study demonstrate that if the EGM recorded immediately after active lead fixation shows an R-wave amplitude > COI magnitude pattern, stable local myocardial amplitude detection is ensured. In these cases, sufficient R-wave amplitude can be anticipated from the onset, and the need for lead repositioning is likely to be minimal. A subsequent rising trend in R-wave amplitude for at least 5 minutes and its stable maintenance are almost guaranteed.

If the EGM immediately after active lead fixation shows an R-wave amplitude < COI magnitude morphology, both implantation success with the Dipper- or Other-type R-wave fluctuations and failure due to low R-wave amplitude would be predicted. In these cases, the final acceptability of the R-wave amplitude depends on whether the true local myocardial amplitude, masked by injury immediately after the implantation, is sufficiently high or not. In cases with an SBT <6 minutes, a Dipper-type trend with a favorable amplitude increase may be predicted and lead repositioning is unlikely to be necessary. However, in cases with SBT over 6 minutes, which suggests the possibility of insufficient local myocardial potential amplitude, lead repositioning may be considered, as the R-wave amplitude fluctuation may present as Dipper- or Other-type patterns without a favorable amplitude increase or could lead to unsuccessful implantation because of low amplitude. SBT within 6 minutes was an independent predictor of favorable amplitude increase in multivariate logistic regression analyses.

Although further investigations are needed, a clinical implication of this study is that a favorable R-wave amplitude increase might be predicted from the EGM morphology obtained immediately after active fixation lead implantation and from the trend in the subsequent 5 to 6 minutes. This finding may lead to a reduction in the number of lead screws, associated complications, and operative time.

### Study limitations

First, this was a single-center study. The relatively small sample size is a limitation of this study. Because the number of patients in the Other-type subgroup was small, the statistical power for between-group comparisons was limited, which may have increased the risk of type II error. Second, as this study examined lead parameters only during the acute intraoperative period, the course of lead parameters during the chronic period remains unknown. Third, although the ROC analysis identified a 6-minute SBT threshold for predicting a favorable R-wave amplitude increase (AUC = 0.73; 95% CI: 0.61-0.85), its specificity was relatively modest (0.61; 95% CI: 0.50-0.83). This suggests that the threshold should not be interpreted as a strict decision boundary, as some cases with adequate final R-wave amplitude may initially appear unfavorable. Nevertheless, the relatively high sensitivity (0.82; 95% CI: 0.58-0.88) indicates that this parameter may still serve as a useful intraoperative indicator to avoid premature lead repositioning without taking an additional waiting time. Fourth, because this study used only the model 3830 leads and Care Link Smart Sync programmer to unify the measurement system, it remains uncertain whether similar findings could be obtained with other leads, programmers, or electrophysiological measurement systems. Further studies are needed to confirm the generalizability of our observations. Fifth, the automatic algorithm for R-wave amplitude sensing of the programmer operates in an obscure manner, making it impossible to provide a definitive explanation for the predicted R-wave fluctuation mechanism in this study. In line with our hypothesis, the mean value of the R-wave amplitude measured by the programmer was higher than that of COI magnitude immediately after the active lead fixation in the Riser type. However, the mean value of R-wave amplitude measured by the programmer was not critically close to that of COI magnitude immediately after the active lead fixation in the Dipper type. Nevertheless, demonstrating specific judgment indicators with clinical implications is considered a significant achievement of the present study.

## Conclusions

More than 97% of the intraoperative R-wave amplitude variation patterns immediately after successful lead implantation could be classified as Dipper or Riser types. The intraoperative R-wave amplitude fluctuation could be predicted from the EGM morphology immediately after RV lead implantation and the subsequent 5- to 6-minute trend.

## Funding Support and Author Disclosures

This research was supported by the Japan Health Research Promotion Bureau (grant number: JH2025WAKATE10). Drs Kusano, Ishibashi, Ueda, and Oka received remuneration for lectures from Medtronic Japan, Inc. All other authors have reported that they have no relationships relevant to the contents of this paper to disclose.
