# Supplementary material for: Association Between Current of Injury Magnitude and Intraoperative R-Wave Amplitude Fluctuation After Pacing Lead Implantation
Source: JACC Asia. 2026 Apr 11;6(8):1472–81. doi: 10.1016/j.jacasi.2026.01.030 (PMC13458809; doi:10.1016/j.jacasi.2026.01.030)
Supplement: Supplementary file 1 [file mmc1.docx]

**Supplemental Figure 1: Alternative electrogram pattern with negative R-wave deflection.** The panels present the negative R-wave deflection pattern bipolar electrograms recorded on the right ventricular lead in a representative Riser-type case.

Abbreviation: EGM, electrocardiogram.

**Supplemental Figure 2: Intraoperative R-wave amplitude fluctuation patterns of the Other-type of successful implantations and failed implantations.**

A: The Other-type R-wave amplitude transitions of successful right ventricular lead implantations. B: The R-wave amplitude transitions in cases where RV implantation failed due to low R-wave amplitude.

The bottom panels show the bipolar electrogram recorded on the right ventricular leads in representative cases. The yellow and green dots indicate the highest amplitude points in each phase.
